# Percutaneous suction and irrigation for the treatment of recalcitrant pyogenic spondylodiscitis

**DOI:** 10.1186/s10195-018-0496-9

**Published:** 2018-08-20

**Authors:** William Griffith-Jones, Luigi Aurelio Nasto, Enrico Pola, Oliver M. Stokes, Hossein Mehdian

**Affiliations:** 1 Exeter Spinal Unit, Princess Elizabeth Orthopaedic Centre, Royal Devon & Exeter NHS Foundation Trust, Barrack Road, Exeter, Devon UK; 2 The Centre for Spinal Studies and Surgery, Queen’s Medical Centre, Nottingham University Hospitals, Derby Road, Nottingham, NG7 2UH UK; 3 Spinal Unit, “A. Gemelli” University Hospital, Catholic University of Rome, L.go Agostino Gemelli 8, Rome, Italy

**Keywords:** Pyogenic spondylodiscitis, Minimally invasive technique, Spinal abscess drainage, Spinal percutaneous drainage

## Abstract

**Background:**

The primary management of pyogenic spondylodiscitis is conservative. Once the causative organism has been identified, by blood culture or biopsy, administration of appropriate intravenous antibiotics is started. Occasionally patients do not respond to antibiotics and surgical irrigation and debridement is needed. The treatment of these cases is challenging and controversial. Furthermore, many affected patients have significant comorbidities often precluding more extensive surgical intervention. The aim of this study is to describe early results of a novel, minimally invasive percutaneous technique for disc irrigation and debridement in pyogenic spondylodiscitis.

**Materials and methods:**

A series of 10 consecutive patients diagnosed with pyogenic spondylodiscitis received percutaneous disc irrigation and debridement. The procedure was performed by inserting two Jamshidi needles percutaneously into the disc space. Indications for surgery were poor response to antibiotic therapy (8 patients) and the need for more extensive biopsy (2 patients). Pre- and postoperative white blood cell count (WBC), C-reactive protein (CRP), erythrocyte sedimentation rate (ESR), Oswestry disability index (ODI), and visual analogue score (VAS) for back pain were collected. Minimum follow-up was 18 months, with regular interval assessments.

**Results:**

There were 7 males and 3 females with a mean age of 67 years. The mean WBC before surgery was 14.63 × 10^9^/L (10.9–26.4) and dropped to 7.48 × 10^9^/L (5.6–9.8) after surgery. The mean preoperative CRP was 188 mg/L (111–250) and decreased to 13.83 mg/L (5–21) after surgery. Similar improvements were seen with ESR. All patients reported significant improvements in ODI and VAS scores after surgery. The average hospital stay after surgery was 8.17 days. All patients had resolution of the infection, and there were no complications associated with the procedure.

**Conclusions:**

Our study confirms the feasibility and safety of our percutaneous technique for irrigation and debridement of pyogenic spondylodiscitis. Percutaneous irrigation and suction offers a truly minimally invasive option for managing recalcitrant spondylodiscitis or for diagnostic purposes. The approach used is very similar to discography and can be easily adapted to different hospital settings.

**Level of Evidence:**

Level III

**Electronic supplementary material:**

The online version of this article (10.1186/s10195-018-0496-9) contains supplementary material, which is available to authorized users.

## Introduction

The term pyogenic spondylodiscitis is used to describe a spectrum of spinal infections encompassing vertebral osteomyelitis, spondylitis and discitis. Although relatively rare, spondylodiscitis is the main manifestation of haematogenous osteomyelitis in patients aged >50 years [1] and represents 3–5% of all cases of osteomyelitis [2, 3]. Estimates of its incidence range from 4−24 per million per year in developed countries [4]. Diabetes is the most common risk factor for the development of spondylodiscitis; other risk factors include advancing age, intravenous drug use, immunosuppression, malignancy, renal failure, rheumatological disease and previous spinal surgery [5]. Although a wide range of organisms have been associated with spondylodiscitis, it remains primarily a monomicrobial bacterial infection with *Staphylococcus aureus* being the predominant pathogen in over half of the reported cases [6]. More than half of all cases of haematogenous spondylodiscitis occur within the lumbar spine followed by the thoracic and cervical spine in decreasing frequency (58, 30 and 11%, respectively) [7].

The primary management of spondylodiscitis is conservative. Once the causative organism has been identified, by blood culture or biopsy, appropriate intravenous (IV) antibiotics are started. Treatment regimens of 6–14 weeks have been reported, with criteria for discontinuation including resolution of symptoms to normalisation of inflammatory markers [6]. Indications for surgical intervention include neural compression, spinal instability and deformity or failure of conservative management [8]. The aim of surgical treatment in cases that are unresponsive to conservative treatment is to provide early debridement of the infection and decrease the bacterial load. Surgery is also indicated in more severe cases when neurology or mechanical stability is compromised.

Historically, debridement and drainage of disc abscess has been performed through open surgery and anterior exposure of the spine. More recently, minimally invasive techniques for surgical management of resistant spondylodiscitis have been described [9–15]. Percutaneous transpedicular discectomy and drainage (PTDD) makes use of the pedicle as the entry point to the disc space [10, 12, 13]. Although this allows safe access to the disc space, the main drawbacks of this technique are limited access to the disc space due to the orientation and limited size of the pedicle as well as the risk of spreading the infection to the posterior elements. Percutaneous endoscopic discectomy (PED) was first developed for the treatment of herniated discs in the 1980s. More recently, this technique has been adapted for use in treating spinal infections [9, 11, 14]. PED allows better access than PTDD and direct visualisation of the disc space; however, it requires skills and equipment only available in centres which routinely carry out PED [15].

The aim of this study was to report on the feasibility of a novel percutaneous technique for irrigation and debridement of the intervertebral disc space in patients with pyogenic spondylodiscitis. Our technique makes use of an approach to the disc space similar to discography and does not require any specific equipment. A detailed description of the technique is provided along with a retrospective analysis of a consecutive series of patients who were successfully treated by the authors.

## Materials and methods

Following institutional review board approval (as part of service evaluation), the records of 10 consecutive patients surgically treated with our newly developed technique for percutaneous drainage of disc abscess from October 2014 to December 2015 were retrieved from our database. All patients had been diagnosed with pyogenic spondylodiscitis. In eight patients, the causative agent had been identified through blood cultures or computed tomography (CT) guided biopsy; however, in two patients no causative agent could be identified. Indications for surgery were failure to respond to antimicrobial therapy (eight patients) and the need of a more extensive biopsy to establish a microbiological diagnosis (two patients). Failure to respond to antibiotic therapy (i.e., poor responders) was defined as a ≤ 50% decrease of CRP levels after a 2-week course of IV antibiotic therapy. All patients were evaluated by our infectious disease (ID) team and received IV antibiotic therapy as directed by the ID team. All patients were discharged home on antibiotic therapy when clinically stable and were seen in the outpatient clinic at 4, 8, 12, and 24 weeks after surgery. White blood cell count (WBC), C-reactive protein (CRP), and erythrocyte sedimentation rate (ESR) levels were measured during hospital admission and at every outpatient follow-up. Oswestry disability index (ODI) and visual analogue scale (VAS) scores were also collected at diagnosis and at each follow-up appointment. The clinical charts of the patients were reviewed for any perioperative complication.

### Surgical technique

**Fig. 1:**
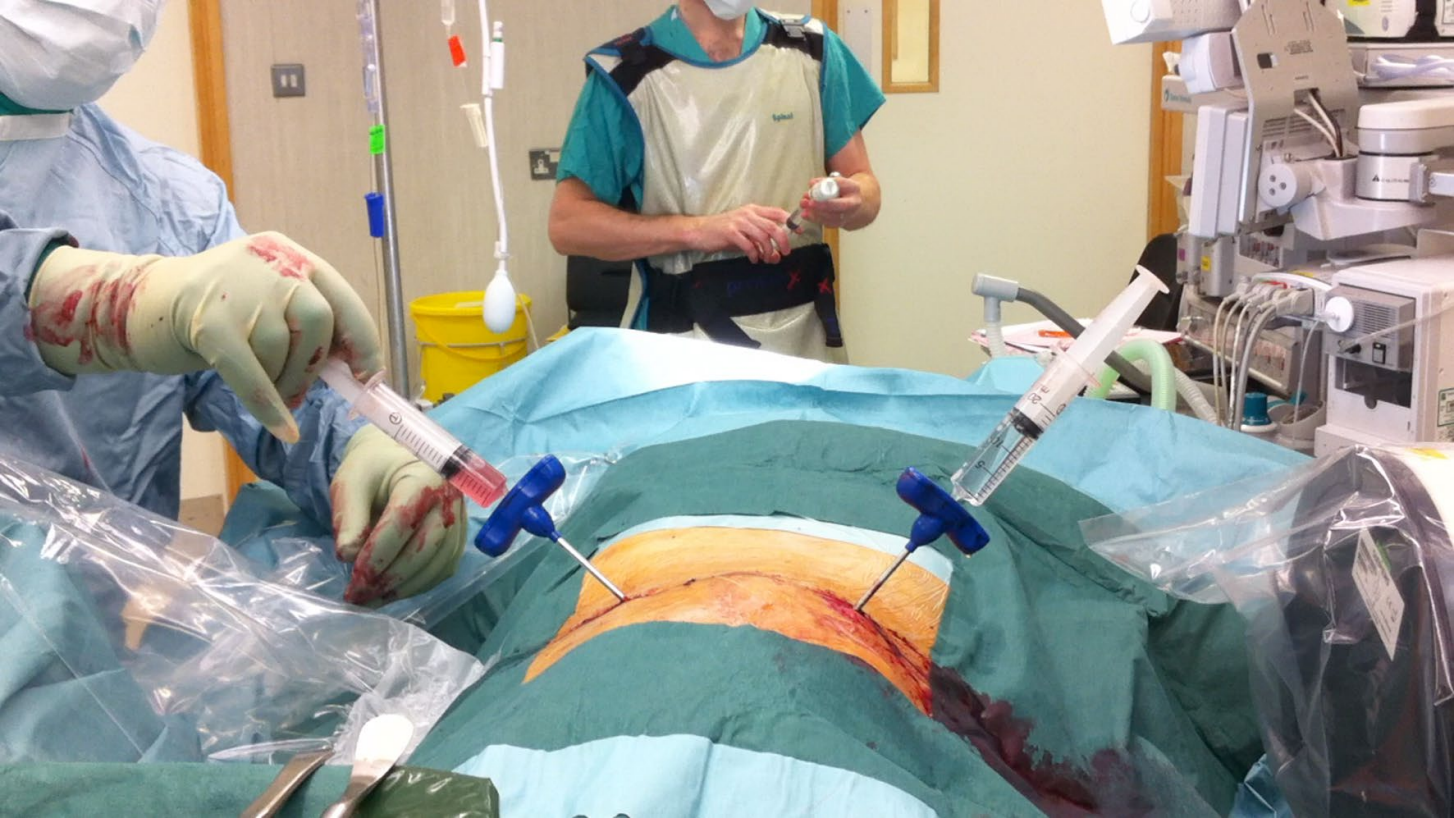
Intraoperative positioning of the patient. The patient is positioned prone on bolsters. The surgical area is draped and a C-arm is positioned perpendicular to the patient for intraoperative monitoring of needle placement

**Fig. 2:**
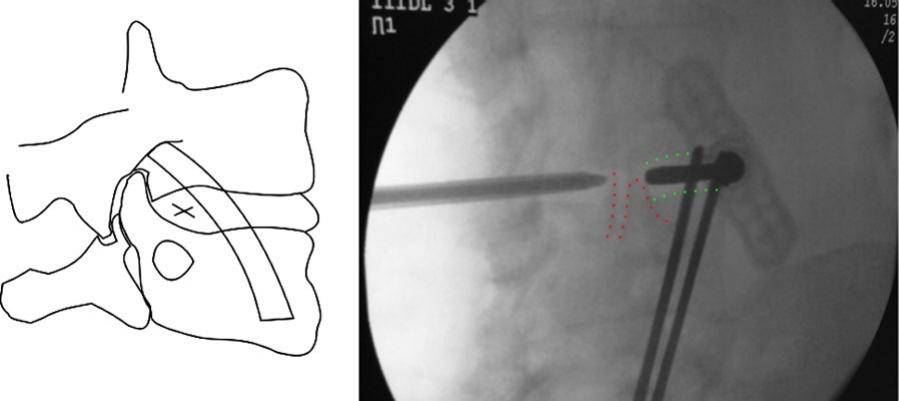
Lordosis of the C-arm is adjusted on the AP view to obtain a perfectly parallel view of the vertebral endplates of the target disc (green dotted lines). Following this, lateral tilt of the C-arm is adjusted to obtain an oblique view of the target disc making sure that the articular facet is correctly visualised in the posterior third of the disc space (red dotted lines). The entry point into the disc is marked on the schematic drawing (left panel)

**Fig. 3:**
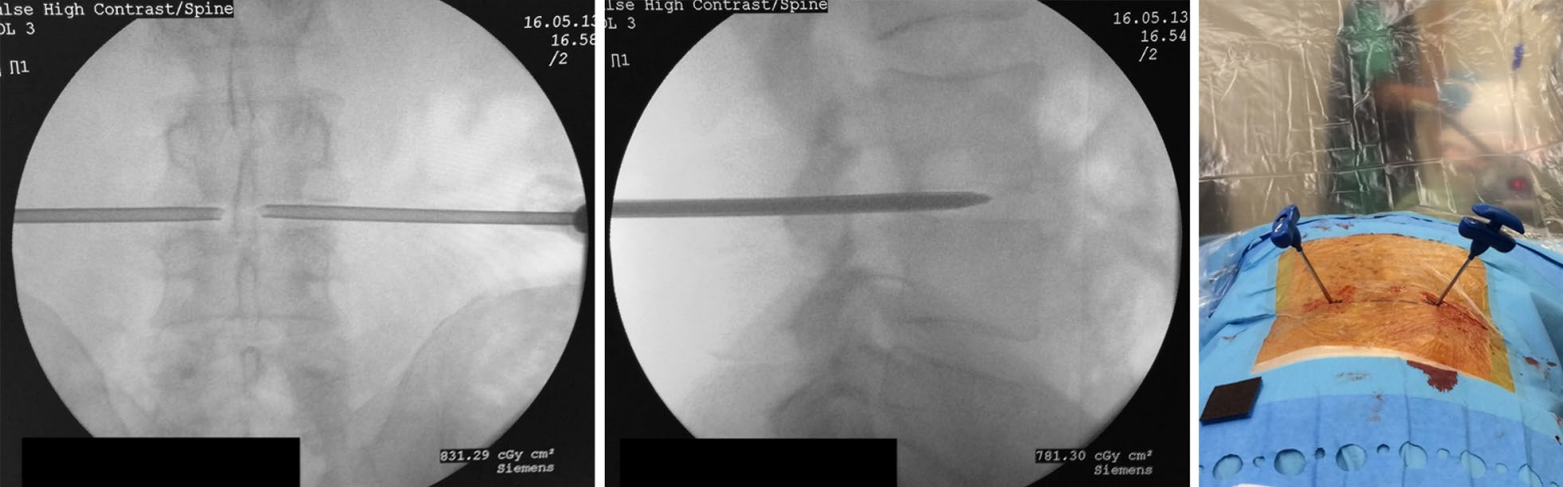
The needles are advanced inside the disc space. (Left panel) final position of the needles in the AP view; (middle panel) final position of the needles in the lateral view; (right panel) intraoperative position of the needles

The surgical procedure can be performed under local anaesthesia and conscious sedation. The patient is positioned prone on a radiolucent table, and the lumbar area is prepared and draped. The C-arm fluoroscope is positioned to obtain a clear view of the disc space to be washed out, which is centralized in the image (Fig. 1). The degree of lordosis on the C-arm is adjusted in order to obtain an optimal image with parallel vertebral endplates of the vertebral body above and below the disc space. Following this, the C-arm is tilted to obtain an oblique view of the disc space until the superior articular process is visualised halfway between the anterior and posterior disc margins (Fig. 2). The target point for needle entry lies just anterior to the superior articular process, along the lower aspect of the disc (to avoid contact with the exiting nerve root). The entry point is identified using a metallic marker on the skin (k-wire) and a Jamshidi needle is inserted along the line of the X-ray beam, into the disc space. In a similar way, a second needle is inserted in the same disc from the contralateral side (Fig. 3). For the L5/S1 disc space the fluoroscope may require significantly more caudal angulation and obliquity in order to obtain a parallel view of the disc endplates. The entry point into the disc in this case is a small triangle formed by the superior articular process posteriorly, the iliac crest anteriorly and the inferior bony border of the L5 vertebral body. A 20-mL syringe is attached to one of the Jamshidi needles and an attempt is made to aspirate the disc content. The sample obtained is sent for microbiological analysis. Following this, a second 20-mL syringe containing normal saline is attached to the second Jamshidi needle. The disc is then washed out with normal saline solution, which is collected in the empty syringe. The first 40 mL of saline disc washout is sent for microbiological evaluation, and the remaining washout fluid is discarded. This process is then repeated and reversed until the fluid runs clear and at least 500 mL of normal saline has been flushed through the disc space (Additional file 1: Video S1).

## Results

A total of 10 patients (7 male and 3 female) underwent percutaneous drainage of disc abscess for pyogenic spondylodiscitis using the method described above over a 14-month period. The mean age was 67 years (51–82 years). The patients were all initially managed with appropriate antibiotics following confirmation of the causative pathogen by blood culture. In two patients, antibiotic therapy was withheld until percutaneous drainage of the disc was performed to obtain adequate samples for diagnosis. *S. aureus* was isolated in seven patients and treatment with IV flucloxacillin and clindamycin was initiated. In the remaining three cases, *Escherichia coli* was isolated and treated with amoxicillin. The mean duration of treatment prior to surgery was 2 weeks (1–6 weeks).

The mean WCC levels were 10.15 × 10^9^/L (7.9–13.1) on presentation and 14.63 × 10^9^/L (10.9–26.4) following treatment with antibiotics [average increase of 4.48 × 10^9^/L (0.6–13.3)], indicating a failure to respond to the therapy. Postoperatively, the average WCC decreased to 7.48 × 10^9^/L (5.6–9.8), i.e., a reduction of 7.15 × 10^9^/L (2.8–16.6). The mean CRP was 301.33 mg/L (173–600) on presentation and decreased to 188 mg/L (111–250) after antibiotic therapy, i.e., a reduction of 113.33 mg/L (29–350). Following percutaneous disc washout, the mean CRP reduced to 13.83 mg/L (5–21), i.e., a mean reduction of 174.16 mg/L (102–229). The mean ESR was 102 mm/h (83–128) preoperatively and reduced to 23.67 mm/h (19–28) following washout of the disc.

**Fig. 4:**
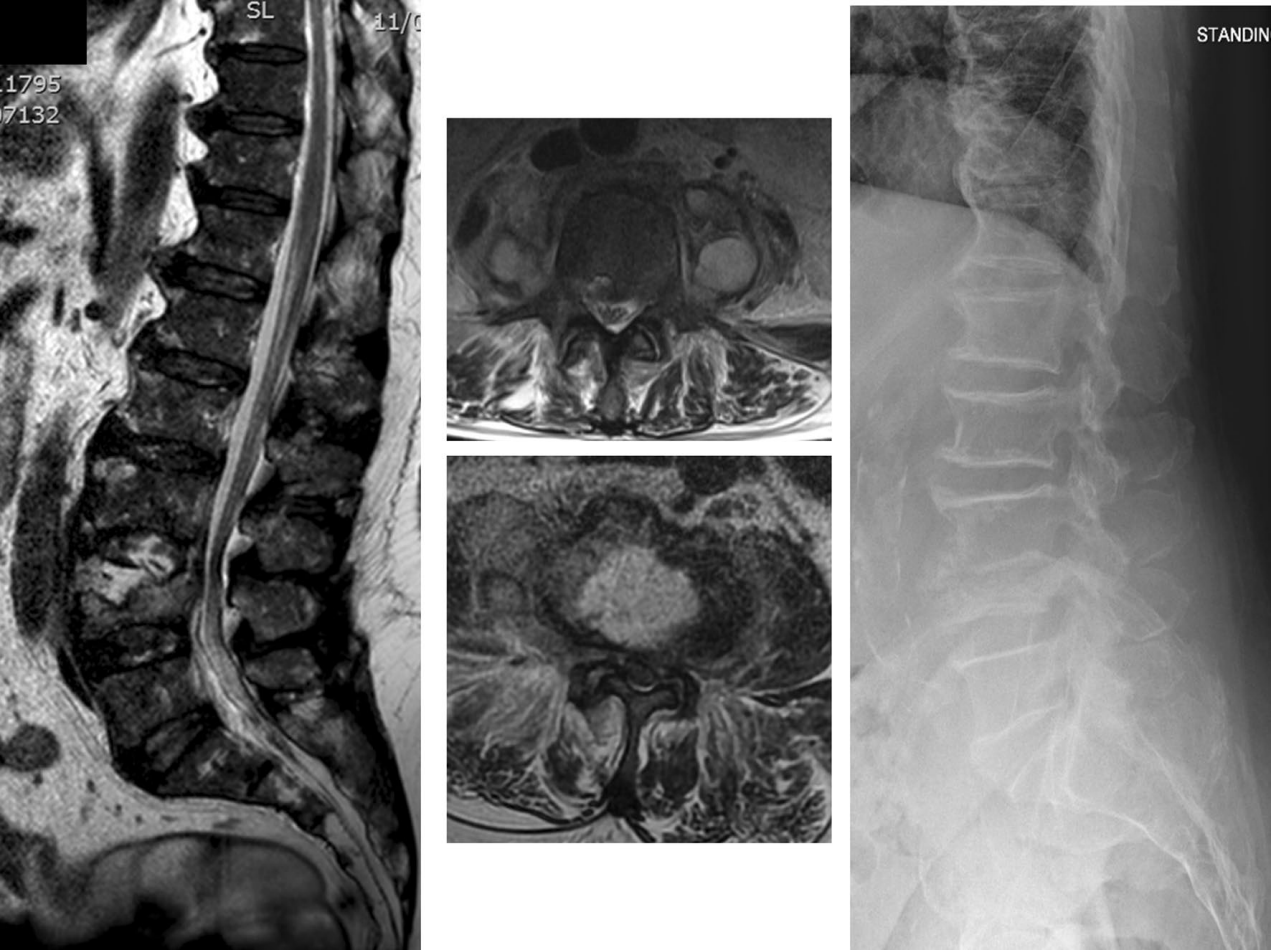
A 75-year-old patient with pyogenic spondylodiscitis at L3/L4. *S. aureus* had been identified by blood cultures 10 days earlier and IV antibiotic therapy was started soon afterwards. After 10 days of IV therapy, the patient was still complaining of significant pain with incomplete improvement of the inflammatory markers (CPR 135 mg/L). (Left panel) MRI sagittal view of the involved disc space; (middle panel) axial view at the level of the L3/L4 disc space and the L4 vertebral body showing a significant disc abscess and bilateral psoas abscesses. (Right panel) standing X-ray of the lumbar spine showing resolution of the infection and fusion of the involved segment 8 months after the end of treatment

The mean length of stay following surgery was 8.17 days (8–12). VAS scores and the ODI were recorded pre- and postoperatively. The mean VAS prior to surgery was 8 (7–9) and postoperatively this reduced to 3 (2–4) on discharge from hospital. The mean ODI was 47.67 (33–58) preoperatively and, following washout of the disc, this reduced to 19 (14–23) on discharge from hospital. There were no complications in this series of 10 consecutive patients. No neurological complications were observed. All patients were treated with a rigid thoraco-lumbo-sacral orthosis brace for 12–16 weeks after discharge from hospital; none of the patients underwent secondary surgical intervention during the study period (Fig. 4).

## Discussion

Spondylodiscitis is a rare condition; however, the incidence is increasing due to a growing at-risk population and improved rates of diagnosis [6]. Medical treatment remains the mainstay of management, with administration of appropriate antibiotics after the causative organism has been isolated. Indications for surgery include neurological compromise, vertebral collapse and failure of conservative management.

Traditional surgical management involved open washout and debridement of infected material [2]. This carried the risk of significant morbidity in a patient group where most of them have pre-existing health problems [5]. As a result, several minimally invasive techniques have been described. These have been shown to yield successful results in the treatment of spondylodiscitis but each have their own limitations [8]. The transpedicular route involves the violation of otherwise generally unaffected posterior elements with potential complications such as fracture and neurological injury. Arya et al. reported a series of 15 patients undergoing transpedicular discectomy and drainage for spondylodiscitis [10]. There were no early or late surgical complications, eight patients experienced an improvement in symptoms within 24 h. Four patients failed to respond and underwent open debridement and fusion. Hadjipavlou et al. described similar management of six patients for treatment of resistant discitis [12]. Five patients reported significant symptomatic relief following the procedure with a follow-up of 18 months. The percutaneous methods previously described are adaptions of techniques used for elective discectomy and, as such, require specialist equipment which may only be available in centres where this is routinely performed [9, 11, 14]. Haaker et al. and Li et al. reported a series of 26 and 34 patients, respectively, where initial organism culture had been identified via percutaneous discectomy [11, 14]. Both series reported no significant postoperative complications. Fu et al. reported 14 patients with pyogenic spondylodiscitis treated by percutaneous endoscopic discectomy and drainage [15]. Two of these patients required early open debridement and anterior fusion and a further two patients had recurrent infection and underwent subsequent open surgery.

In this report, we describe a novel technique for percutaneous drainage of disc abscess in pyogenic spondylodiscitis. Our technique makes advantage of a similar approach to that used for discography. As such, only a C-arm image intensifier and two Jamshidi needles are needed. This procedure can be performed under local anaesthesia or conscious sedation. It can be used for diagnostic purposes (when blood cultures or CT-guided biopsy are negative) or to decrease the infection burden and debride the disc space in non-responsive cases. In our series, the procedure was used in patients with a ≤ 50% decrease of CRP level after a 2-week course of IV antibiotics. The procedure allows quick and minimally invasive access to the disc space. The access to the disc space through soft tissue allows more freedom to the needle and better access to the disc space. Furthermore, this avoids penetration of the bone elements of the posterior arch and the spinal canal, reducing the risk of posterior spread of the infection. The access to the disc allows debridement of the disc space and decrease of the infection burden. In this way, hypoxic and poorly perfused areas of the disc can be accessed and local antibiotics can be injected at the end of the procedure. Finally, this technique is technically less demanding and can be easily adapted to different hospital settings. Although we have not noticed any complication in a first series of 10 patients, we would caution against the use of this technique in cases with severe compromise of the posterior annulus to avoid spread of the infection to the posterior canal. We believe our technique may be a valuable adjunct to the minimally invasive treatment of spinal infections and should be considered in patients with early stage spinal infections or serious medical conditions. Our technique is not indicated in cases were mechanical stability of the spine is compromised or if there is any risk of impending neurological compromise.

## Additional file

**Additional file 1.** Intraoperative drainage of the disc abscess through the two Jamshidi needles inserted in the disc space.
